# *Ganoderma tsugae* Induces S Phase Arrest and Apoptosis in Doxorubicin-Resistant Lung Adenocarcinoma H23/0.3 Cells via Modulation of the PI3K/Akt Signaling Pathway

**DOI:** 10.1155/2012/371286

**Published:** 2012-06-26

**Authors:** Yang-Hao Yu, Han-Peng Kuo, Hui-Hsia Hsieh, Jhy-Wei Li, Wu-Huei Hsu, Shih-Jung Chen, Muh-Hwan Su, Shwu-Huey Liu, Yung-Chi Cheng, Chih-Yi Chen, Ming-Ching Kao

**Affiliations:** ^1^Graduate Institute of Clinical and Medical Science, School of Medicine, China Medical University, Taichung 40402, Taiwan; ^2^Division of Pulmonary and Critical Care, China Medical University Hospital, Taichung 40447, Taiwan; ^3^Department of Biological Science and Technology, College of Life Sciences, China Medical University, Taichung 40402, Taiwan; ^4^Pharmacy Department, China Medical University Hospital, Taichung 40447, Taiwan; ^5^Department of Pathology, Da-Chien General Hospital, Miaoli 36052, Taiwan; ^6^Luo-Gui-Ying Fungi Agriculture Farm, Taoyuan 33043, Taiwan; ^7^Sinphar Group R&D Center, Yilan 26944, Taiwan; ^8^Division of Drug Development, PhytoCeutica, Inc., New Haven, CT 06511-1989, USA; ^9^Department of Pharmacology, Yale University School of Medicine, New Haven, CT 06520-8066, USA; ^10^Division of Chest Surgery, China Medical University Hospital, Taichung 40447, Taiwan; ^11^Department of Biochemistry, National Defense Medical Center, Taipei 11490, Taiwan

## Abstract

*Ganoderma tsugae* (GT) is a traditional Chinese medicine that exhibits significant antitumor activities against many types of cancer. This study investigated the molecular mechanism by which GT suppresses the growth of doxorubicin-resistant lung adenocarcinoma H23/0.3 cells. Our results reveal that GT inhibits the viability of H23/0.3 cells *in vitro* and *in vivo* and sensitizes the growth suppression effect of doxorubicin on H23/0.3 cells. The data also show that GT induces S phase arrest by interfering with the protein expression of cyclin A, cyclin E, CDK2, and CDC25A. Furthermore, GT induces cellular apoptosis via induction of a mitochondria/caspase pathway. In addition, we also demonstrate that the suppression of cell proliferation by GT is through down-regulation of the PI3K/Akt signaling pathway. In conclusion, this study suggests that GT may be a useful adjuvant therapeutic agent in the treatment of lung cancer.

## 1. Introduction

Lung carcinoma is the most predominant form of cancer and has surpassed breast carcinoma as the leading cause of cancer mortality in the United States; it accounts for approximately 26% and 28% of all female and male cancer deaths, respectively [1]. Lung carcinoma was also the leading cause of cancer death in Taiwan for women and men in 2010 [2]. Moreover, an apparent increasing death rate of lung carcinoma from 1986 to 2010 has also been observed in Taiwan [2]. The two most aggressive forms of lung cancer are non-small-cell lung cancer (NSCLC) and small-cell lung cancer (SCLC), which account for approximately 85% and 15%, respectively, of all lung cancers [3]. Both forms of lung cancer frequently cause drug resistance leading to poor survival [4]. Therefore, alternative medicines and more treatment modalities to overcome drug resistance and to improve the patients' outcomes of this serious disease are urgently desired.

*Ganoderma*, a traditional Chinese medicine (TCM), has been widely used for medicinal purposes in oriental countries for centuries. *Ganoderma lucidum* (GL) and *Ganoderma sinense* (GS), listed as Lingzhi in China pharmacopeia, are two of the most representative species of *Ganoderma* and have a long history of use in folk medicine in China. The biological activities of GL and GS, especially their immunomodulatory and antitumor properties, have been well documented [5]. In addition, *Ganoderma tsugae* (GT), another well-cultivated species of *Ganoderma*, has been investigated and found to possess many biological and pharmacological properties, such as antiinflammation [6], antifibrosis [7], antiautoantibody formation [8], and antioxidation [9]. A number of reports show that GT possess growth inhibition effects on a variety of tumor cells, such as sarcoma 180 cells [10], breast cancer MDA-MB-231 and MCF-7 cells [11], hepatoma Hep3B cells [12], and colorectal cancer COLO 205 cells [13]. Moreover, GT also exerts anti-angiogenesis effects on epidermoid carcinoma A431 cells by modulating the EGFR/PI3K/Akt/mTOR signaling pathway [14]. Although GT exhibits anticancer activities in many human cancer cells, the molecular mechanisms that govern its inhibitory effect on the growth of lung cancer cells are still not clear and need to be explored.

The quality and quantity of TCMs, including *Ganoderma*, are potentially influenced by many factors, including the cultivation methods, the cultivated regions, the growth conditions, the processing procedures, and the formulated preparations [15]. Therefore, the quality control of TCMs must be established scientifically in terms of both the chemical and biological aspects; but to date, this has not been achieved. A newly established and actively progressed Chinese medicine-based academic organization, called the Consortium for Globalization of Chinese Medicine (CGCM; http://www.tcmedicine.org/), is promoting and requesting the quality control of TCMs and also striving to explore the functional use of herbonomics.

In this study, we provide a quality assured ethanol extract of GT (GTE) and demonstrate its anticancer effects and related molecular mechanisms in doxorubicin-resistant NSCLC H23/0.3 cells *in vitro* and i*n vivo*. Our results indicate that the GTE inhibits cellular growth and induces S phase arrest and apoptosis by modulating the PI3K/Akt signaling pathway. Furthermore, we also show that GTE sensitizes H23/0.3 cells to doxorubicin, indicating a potential use of GTE in the treatment of lung cancer with drug resistance.

## 2. Materials and Methods

### 2.1. Cell Culture

Two non-small-cell lung cancer (NSCLC) cell lines were used in this study. H23 (CRL-5800, ATCC), a lung adenocarcinoma cell line, was purchased from ATCC (American Type Culture Collection). H23/0.3, a doxorubicin-resistant H23 cell line, was a gift from Dr. Chun-Ming Tsai (Department of Chest, Veterans General Hospital, Taipei, Taiwan); the cell line was generated by the stepwise exposure of H23 to increasing concentrations of doxorubicin up to 0.3 *μ*g/mL. All cells were cultured in RPMI-1640 medium (Gibco BRL) supplemented with 10% fetal bovine serum in a humidified atmosphere with 5% CO_2_ at 37°C.

### 2.2. Chemicals and Antibodies

The anticytochrome c antibody and propidium iodide (PI) were obtained from Sigma-Aldrich Co. (St. Louis, MO, USA). The antibodies against cyclin A, cyclin E, Akt1, Bax, and caspase-3 were purchased from Santa Cruz Biotechnology (Santa Cruz, CA, USA). The anti-PARP antibody was obtained from Biovision Inc. (Mountain View, CA, USA). The antibodies against Bcl-2, CDK2, CDC25A, CDC25B, and CDC25C were purchased from Abcam, Inc. (Cambridge, MA, USA). The anti-phospho-Akt (Thr473) antibody, the horseradish peroxidase-conjugated anti-rabbit and anti-mouse IgG antibodies, and the PI3K inhibitor (LY294002) were purchased from Cell Signaling Technology, Inc. (Beverly, MA, USA). The anti-*β*-actin antibody was purchased from Chemicon International, Inc. (Temecula, CA, USA). The IRDye 800-conjugated affinity purified anti-rabbit and anti-mouse IgGs were purchased from Rockland Immunochemicals, Inc. (Gilbertsville, PA, USA). Doxorubicin was purchased from Pharmacia (Pharmacia & Upjohn S.P.A. Milan, Italy).

### 2.3. Preparation of *Ganoderma tsugae* Extracts

*Ganoderma tsugae* (GT) was kindly provided by the Luo-Gui-Ying Fungi Agriculture Farm (with a registered name of Tien-Shen Lingzhi), Taoyuan, Taiwan. Briefly, the powder of the GT fruiting body (20 g) was soaked in 99.9% ethanol (400 mL), mixed, and shaken for 24 h with a rotating shaker. After centrifugation, the supernatant was filtered through filter paper (Whatman, Cat. No. 1001-110), and the residues were extracted with alcohol two additional times as mentioned above. The filtrates were collected and subjected to concentration under reduced pressure (i.e., evaporated to dryness under reduced pressure) to produce a brown gel-like GT extract (GTE). The yield was approximately 10%. The GTE was then prepared as a stock solution with ethanol solvent (200 mg/mL) and stored at −20°C until use.

### 2.4. High-Performance Liquid Chromatography (HPLC)

Sample preparation: the sample was diluted, by the addition of ethanol to 1.0 mg/mL, and filtered through a 0.2 *μ*m Millipore filter; then, 10 *μ*L of the sample was subjected to HPLC analysis. Sample separation: the sample (10 *μ*L) was automatically delivered into a C-18 column (LiChroCART, 250 × 4.6 mm, 5 *μ*m, Merck, Germany) for separation via an HPLC system (L-7100 pump and L-7400 UV-vis detector, Hitachi, Tokyo, Japan). The initial mobile phase comprised acetonitrile (30%) and phosphoric acid (0.11%, pH 2.2) with a flow rate of 1 mL/min; the percentage of acetonitrile in the mobile phase was increased from 30% to 100% in a linear gradient for the first 20 minutes and then maintained at 100% until the end of the experiment. The detection of signals was set at wavelength of 288 nm.

### 2.5. Electrospray Ionization Mass Spectrometry (ESI-MS)

GTE was dissolved in ethanol (10 ng/*μ*L), and the resulting solution was directly injected into the ion source (Esquire HCT ultra PTM, Bruker) using ESI (positive mode) at a flow rate of 240 *μ*L/min. The mass range was acquired from 100 to 1,000 m/z.

### 2.6. Quality Control of GTEs via Bioresponse Fingerprinting

To maintain and assess the quality of the GT extracts (GTEs), a comprehensive platform for quality control of botanical drugs, named PhytomicsQC [16], was used in this study. In addition to the chemical fingerprinting method (e.g., HPLC & ESI-MS) as mentioned above, a biological response fingerprinting method from the PhytomicsQC technology [16] was also performed in this study. Briefly, the bioresponse is measured by defining the set of genes that are significantly regulated in cell cultures treated with the GTE. We used optimized standard operating procedures at every step, including cell banking, tissue culture, botanical extractions, cell culture treatments, and RNA extractions. The RNA is used to obtain transcription profiles in GeneChip hybridization studies using Affymetrix technology. The changes in the individual gene expression levels obtained by the GeneChip experiments were measured by Affymetrix MAS 5.0 software. A statistical pattern comparison method from the PhytomicsQC platform, phytomics similarity index (PSI), was applied to determine the batch-to-batch similarity of the botanical products. In general, clinically similar batches have a PSI of 0.95 or more. The genomic bioresponse to the GTEs was determined in H23/0.3 cells treated with a single IC_50_ dose of GTE for 24 h. The total RNA was extracted from the GTE-treated cells and cleaned with a commercial kit (Qiagene RNA extraction kit, cat# 75144). The quality of the GTEs was then assessed, and the samples were submitted to the National Yang-Ming University Genome Core Laboratory (Taipei, Taiwan) for GeneChip Hybridization experiments. These experiments were repeated independently in duplicate.

### 2.7. Cell Proliferation Assay

Cell proliferation was determined using the MTT metabolic assay as described previously [17]. Briefly, cells were plated onto 96-well microtiter plates (1 × 10^3^–1 × 10^4^ cells/well, depending on the cancer cells used). After the cells adhered to the plates, various doses of GTE were applied to the cells, and the cells were incubated at 37°C for 72 h. At the end of the GTE treatment, the media were aspirated, and the cells were incubated for 4 h in fresh media containing MTT reagent (0.5 mg/mL). Finally, the solution was measured spectrophotometrically at 545 nm against a reference wavelength of 690 nm.

### 2.8. Flow Cytometric Analysis

For the analysis of the cell cycle, the phase distribution was detected by flow cytometry as described previously [17]. After the GTE treatment, the cells were trypsinized, washed with PBS, and fixed with 75% ethanol overnight at −20°C. The fixed cells were washed with PBS and treated with a working solution of propidium iodide (PI) (50 *μ*g/mL PI in PBS plus 1% Tween-20 and 10 *μ*g RNase) for 30 min in the dark at room temperature to stain the cells for subsequent analysis. The DNA contents were measured using flow cytometry (BD FACS Canto). The cell cycle distribution was analyzed with the FCS Express v2.0 software. For the analysis of apoptosis, the cells were stained using the Annexin V-FITC Apoptosis Detection Kit I (BD Biosciences, San Diego, CA) according to the manufacturer's recommendation. The amount of apoptotic cells was determined by flow cytometry (BD FACS Canto) and analyzed by the FCS Express v2.0 software.

### 2.9. Immunofluorescence Microscopy

H23/0.3 cells (2 × 10^5^ cells) were treated with GTE for 24 h and washed with cold DPBS. Then, the Annexin V-FITC Apoptosis Detection Kit I reagent was added to the cells according to the manufacturer's protocol and incubated for 15 min at room temperature in the dark. Photomicrographs were obtained with a Leica TCS SP2 Confocal Spectral Microscope [18]. We also used a fluorescence microscope (Leica DMR) to identify the fragmented and condensed nuclei that were stained with DAPI (4′,6′diamidino-2-phenylindole).

### 2.10. Western Blot Analysis

To determine the changes at the protein level after the GTE treatment, the cells were lysed in RIPA buffer (50 mM Tris-HCl, pH 7.2, 150 mM EDTA, 1% Nonidet P-40, 0.05% SDS, 1 mM PMSF and 1 mM leupeptin). The cell lysates were centrifuged at 14,000 × g for 10 min at 4°C. The supernatant was analyzed by SDS-PAGE and blotted onto a polyvinyldenefluoride (PVDF) membrane. The membrane was subsequently incubated with the specified primary antibody and then incubated with the HRP-conjugated or the IRDye 800-conjugated secondary antibody. Reactive signals were visualized with an Enhanced Chemiluminescence Kit (Amersham Biosciences, Arlington Heights, IL) or the Odyssey Infrared Imaging System (LI-COR Biosciences, Cambridge, UK).

### 2.11. Release of Cytochrome c

The release of cytochrome c (Cyt-c) from the mitochondria to the cytosol was detected as described previously [17]. Briefly, the cells were gently lysed in lysis buffer (1 mM EDTA, 20 mM Tris-HCl, pH 7.2, 250 mM sucrose, 1 mM dithiothreitol, 1.5 mM MgCl_2_, 10 mM KCl, 10 *μ*g/mL leupeptin, 5 *μ*g/mL pepstatin A, and 2 *μ*g/mL aprotinin). The cell lysates were centrifuged at 12,000 × g at 4°C for 10 min to obtain the pellets (the fractions that contained mitochondria) and the supernatants (cytosolic extracts free of mitochondria). The protein content of the supernatant was determined by the Bio-Rad protein assay kit. The protein (20 *μ*g) was resolved by SDS-PAGE (14%) and then transferred onto PVDF membranes for the detection of Cyt-c.

### 2.12. Animal Experiments

The animal experiments were performed as described previously [13] with slight modifications. Briefly, 1 × 10^6^ H23/0.3 cells were subcutaneously implanted into the flank region of nude mice. In total, 26 mice were enrolled in this experiment, 20 tumor-implanted mice were treated with (*n* = 10) and without (*n* = 10) GTE, respectively, and the remaining 6 mice were neither tumor-implanted nor GTE-treated and were used as a reference control. The GTE-treated mice were fed with GTE daily at a dose of 100 mg/kg body weight; this feeding schedule was initiated when the developed tumor was approximately 50 mm^3^ in volume (usually 2-3 weeks after the cancer cells were implanted). The tumor volume and body weight were measured every 3 days. The mice were sacrificed for pathological examination when the tumor volume exceeded 2,000 mm^3^. The tumors were then completely excised from the subcutaneous tissue and weighed. Biochemical and hematological measurements were evaluated for the toxicity test of the drug.

### 2.13. Immunohistochemical Staining

Fourteen H23/0.3-xenografted tumors (seven for each of the GTE-free and GTE-treated groups) and the surrounding mouse tissues were completely excised, fixed in 10% neutral buffered formalin, embedded in paraffin, and sliced for hematoxylin and eosin (H&E) staining to measure the extent of mitotic and necrotic figures. The preparation of samples for H&E staining was performed as described previously [13]. Five high-power fields (5 HPFs, 400X) of H&E-stained slides were counted using the image selection function of Adobe Photoshop, Version 7.0 (Adobe Systems, CA). The number of mitotic figures was counted in 5 HPFs of H&E-stained, necrosis-free areas.

### 2.14. Statistical Analysis

For statistical analysis, the items of cell death percentage and mitoses were calculated with the Mann-Whitney *U* test. The differences in tumor growth were determined by multiple response models, and the tumor weights were compared by the two sample *t*-test. Statistical Products and Services Solution software (SPSS, version 10.0, SPSS UK Ltd., Woking, Surrey, UK) was used for the analysis. Significant levels were set as *P* < 0.05.

## 3. Results

### 3.1. Quality Control of GTE Using Chemical and Bioresponse Fingerprint Analyses

**Figure 1:**
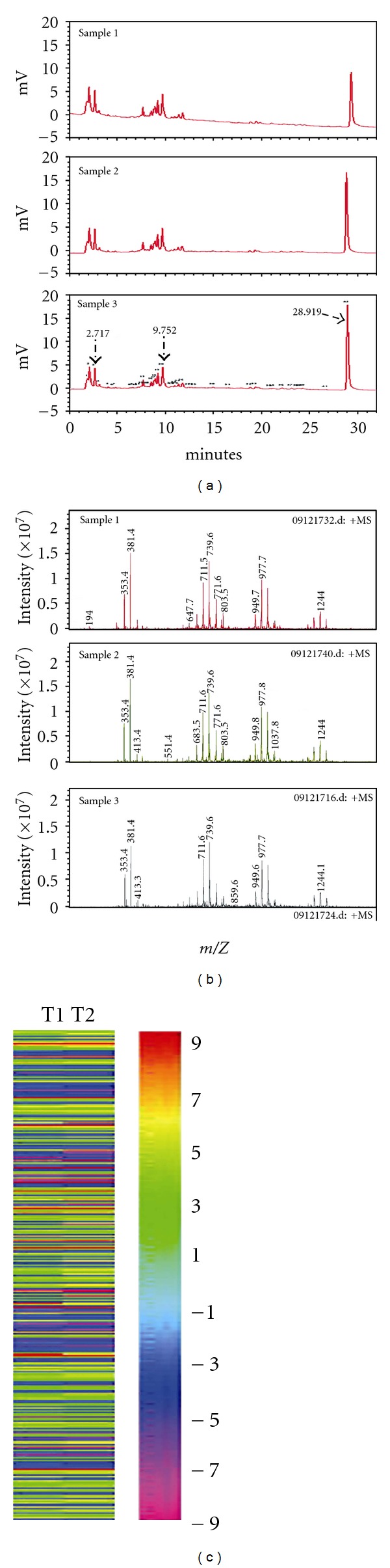
Quality control of GTE. (a) The 3 GTE samples were derived from the fruiting bodies of an improved strain of *Ganoderma tsugae* that were collected at three different times. The HPLC chromatogram shows all of the peaks of the components that eluted before 32 minutes. The chemical fingerprints were found to be identical for the 3 batches, and the specific retention time was given in one example. (b) The identical mass fingerprints were also confirmed using ESI-MS. (c) The biological responses for GTE acting on H23/0.3 analyzed by Phytoviewer QC using the whole genomic approach in duplicate experiments (T1, T2) show highly concordant bioresponse fingerprints with a PSI value of 0.98.

Three batches of GT fruiting bodies that were collected from the same fungi farm at different times were extracted with ethanol. The chemical profiles of the GTEs were analyzed using high-performance liquid chromatography (HPLC). The fingerprints of the 3 batches of GTE were almost identical in triplet experiments. Three representative fingerprints located at 2.717 minutes, 9.752 minutes, and 28.919 minutes were indicated based on their retention times (Figure 1(a)). The last part of the chromatogram corresponded with the major compounds of GTE and had a peak area of 47% (the specific elution time and peak area are based on one representative sample, sample 2). We further verified the chemical profiles of the GTEs with electrospray ionization mass spectrometry (ESI-MS). Similarly, the MS fingerprints for the 3 batches of samples were also indistinguishable (Figure 1(b)). In addition, the bioresponse fingerprints were analyzed by the pattern comparison method of the PhytomicsQC platform, which showed highly concordant biological responses for GTE acting on H23/0.3 cells with a PSI value of 0.98. Under this PSI value, the bioresponse fingerprints contain 338 specifically altered expressed genes with 178 upregulations and 160 downregulations (Figure 1(c)). These results suggest that the GT powder products used in this study have stable, consistent, and high quality.

### 3.2. GTE Suppresses the Growth of Doxorubicin-Resistant H23/0.3 Cells

**Figure 2:**
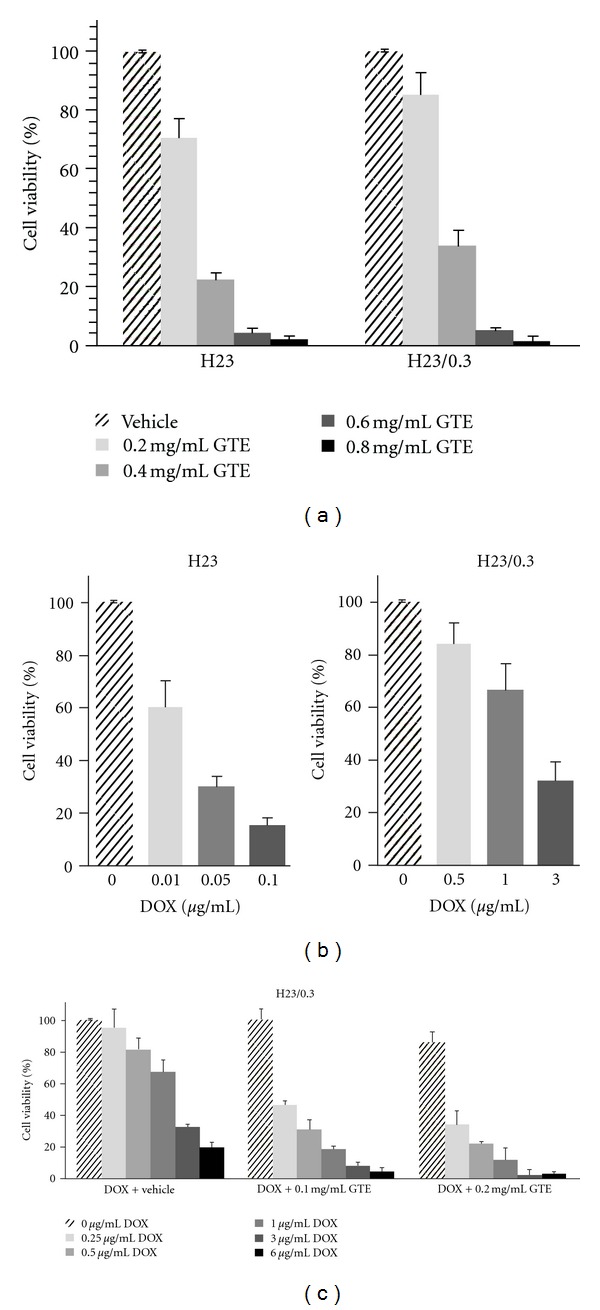
Effect of GTE on cell viability in doxorubicin-resistant lung adenocarcinoma H23/0.3 cells. (a) H23 and H23/0.3 cells were treated with either vehicle control or GTE (0.2 mg/mL, 0.4 mg/mL, 0.6 mg/mL, or 0.8 mg/mL) for 72 h. Cell viability was determined using the MTT assay as described in the Section 2. (b) H23 and H23/0.3 cells were treated with various concentrations of doxorubicin (0.01–0.1 *μ*g/mL in H23 cells; 0.5–3 *μ*g/mL in H23/0.3 cells) for 72 h. Cell viability was determined using the MTT assay. (c) H23/0.3 cells were treated with various concentrations of doxorubicin (0 *μ*g/mL, 0.25 *μ*g/mL, 0.5 *μ*g/mL, 1 *μ*g/mL, 3 *μ*g/mL, and 6 *μ*g/mL) with or without GTE (0.1 mg/mL or 0.2 mg/mL) for 72 h. Cell viability was determined by the MTT assay. Results are expressed as the mean ± SD of three independent experiments.

 To ascertain whether GTE inhibits the growth of lung adenocarcinoma H23 and H23/0.3 cells, we first determined the viability of cells exposed to GTE using the MTT assay. As shown in Figure 2(a), the GTE treatment resulted in a dose-dependent inhibition of cell viability, accounting for a 28–97% and 5–98% reduction in the number of viable cells after treatment with various concentrations of GTE (0.2–0.8 mg/mL) for 72 h in H23 and H23/0.3 cells, respectively. The IC_50_ of GTE was approximately 0.29 mg/mL for H23 cells and 0.34 mg/mL for H23/0.3 cells. The results suggest that GTE is capable of suppressing the proliferation of lung adenocarcinoma H23 and H23/0.3 cells with no selectivity on either cell.

Resistance to anticancer drugs (e.g., doxorubicin and cisplatin) is a major problem in the treatment of patients with lung cancer [19]. We, therefore, examined whether GTE could enhance/sensitize the growth inhibition effects of the anticancer drug doxorubicin in the doxorubicin-resistant H23/0.3 cells (Figure 2(b)), by incubating that cell line with both doxorubicin and GTE. As illustrated in Figure 2(c), GTE significantly enhanced/sensitized the growth suppression effect of doxorubicin on H23/0.3 cells. We found that the number of viable cells was reduced by 14% in cells exposed to GTE (0.2 mg/mL) alone, by 18% in cells exposed to doxorubicin (0.5 *μ*g/mL) alone, and by 78% in cells exposed to both agents combined. Similarly, we also discovered that GTE could enhance the chemotherapeutic efficacy of anticancer drugs against other lung cancer cell lines, for example, H23, A549, and CL1-0 (our unpublished data). These results clearly demonstrate that GTE is able to chemosensitize lung cancer cells to anticancer drugs such as doxorubicin.

### 3.3. GTE Induces S Phase Arrest by Modulating the Expression of Cell Cycle Regulatory Proteins

**Figure 3:**
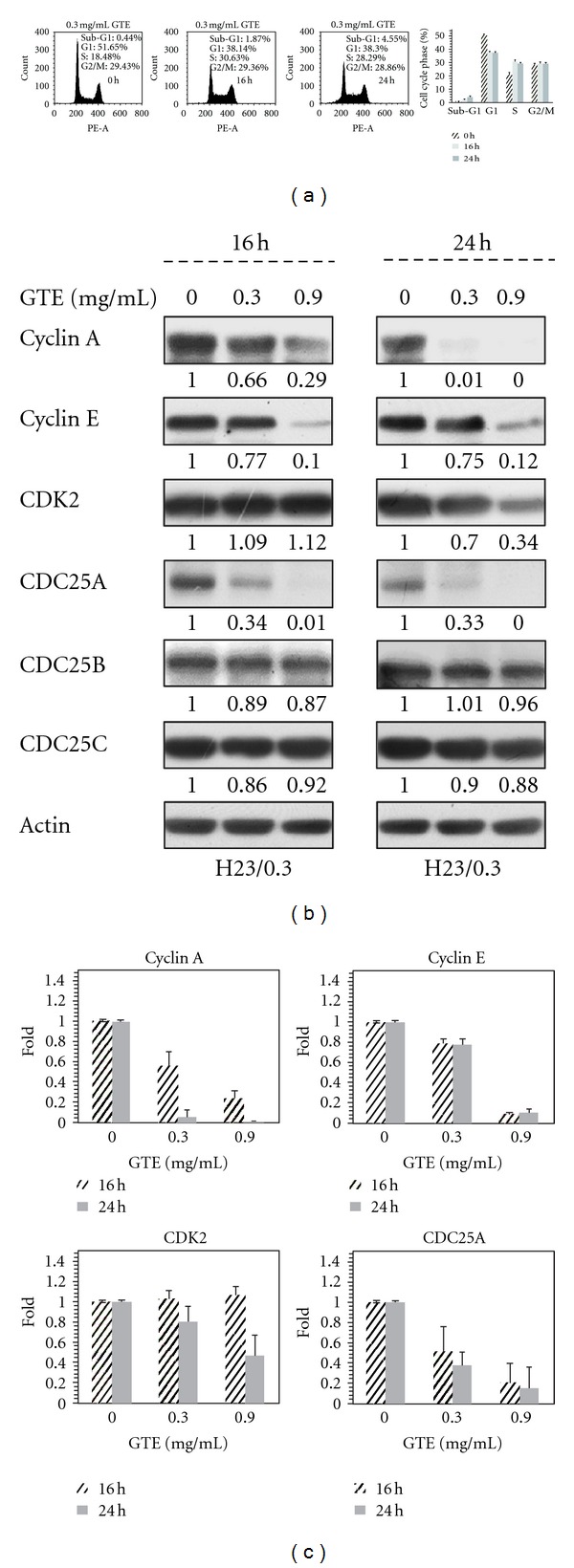
Effect of GTE on cell cycle distribution in H23/0.3 cells. (a) H23/0.3 cells were treated with GTE (0.3 mg/mL) for 16 and 24 h. The cell cycle distribution was measured by flow cytometry as described in Section 2. The error bars indicate the standard deviations of each phase (%), which were the means of three independent experiments. (b) H23/0.3 cells were treated with various concentrations of GTE (0 mg/mL, 0.3 mg/mL, and 0.9 mg/mL) for 16 h and 24 h. The expression of S phase regulators was determined by Western blotting as described in Section 2. (c) A histogram showing the relative protein levels from (b). Results are expressed as the mean ± SD of three independent experiments.

As mentioned above, our results showed a growth suppression effect of GTE on H23/0.3 cells (Figure 2(a)). To verify that the growth inhibition effect of GTE was due to the disruption of the cell cycle, flow cytometry was used to analyze the cell cycle distribution of H23/0.3 cells. As shown in Figure 3(a), the GTE treatment resulted in a marked cell cycle arrest at S phase, which accounted for 12% and 10% increases in the numbers of S phase cells after 16 h and 24 h of treatment with GTE, respectively. This increase in the population of S phase cells was accompanied by an attendant decrease in the G1 phase cell population. These findings suggest that GTE suppresses the growth of H23/0.3 cells by modulating the progression of the cell cycle.

Different regulatory proteins, such as cyclins, cyclin-dependent kinases (CDKs), and cell division cycle 25 (CDC25), work in multiple pathways to tightly modulate the progression of the cell cycle [20]. To identify the molecular mechanisms that govern the GTE-induced S phase arrest, we assessed the effect of GTE on the expression of cell cycle regulators involving in S phase progression. We found that treatment with GTE had a marked dose- and time-dependent inhibitory effect on the protein expression of cyclin A, cyclin E, CDK2, and CDC25A (Figures 3(b) and 3(c)). However, GTE treatment did not cause significant changes in the protein levels of CDC25B and CDC25C (Figures 3(b) and 3(c)). These results suggest that GTE induces S phase cell cycle arrest by modulating the protein expression of cell cycle regulatory proteins in H23/0.3 cells.

### 3.4. GTE Induces Cellular Apoptosis

**Figure 4:**
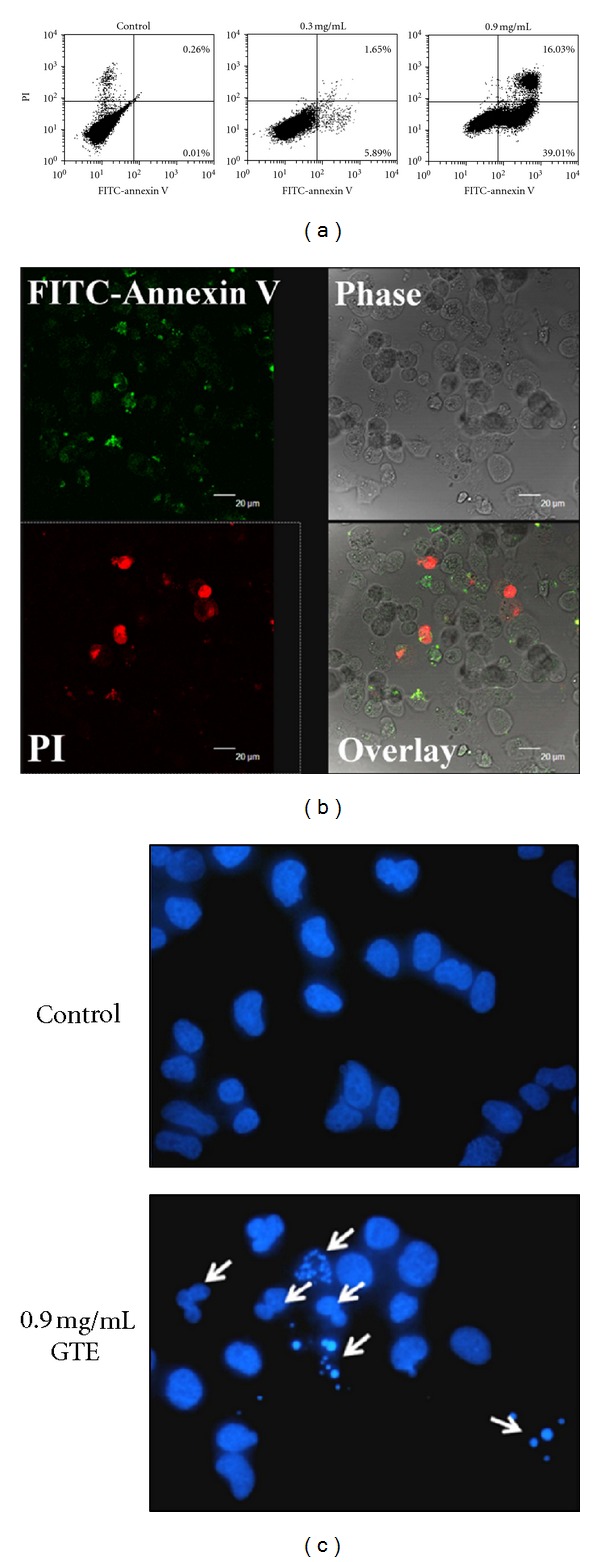
Effect of GTE on cellular apoptosis in H23/0.3 cells. (a) H23/0.3 cells were treated with various concentrations of GTE (0 mg/mL, 0.3 mg/mL, and 0.9 mg/mL) for 24 h. Cellular apoptosis was measured by flow cytometry using the FITC-conjugated annexin V and PI double stains as described in Section 2. (b) H23/0.3 cells were treated with 0.9 mg/mL of GTE for 24 h. The exposure of phosphatidylserine (PS) was measured by confocal microscopy with annexin V-FITC (green color, stained for early asymmetry membrane) and PI (red color, stained for nuclear chromosome after cell membrane disruption) double stains. (c) H23/0.3 cells were treated with 0.9 mg/mL of GTE for 24 h. Fragmented condensed nuclei were measured using a DAPI staining assay.

The suppression of cell proliferation can be achieved by inhibiting cell cycle progression or by inducing cellular apoptosis [21, 22]. To further determine if GTE also induced cellular apoptosis, we analyzed the percentages of apoptotic cells by flow cytometry in H23/0.3 cells following staining with annexin V-FITC and PI. Apoptotic cells were shown in the upper right (as late apoptotic cells) or lower right (as early apoptotic cells) quadrants of the FACS histogram. We found that the treatment of H23/0.3 cells with 0.9 mg/mL of GTE resulted in a marked induction of apoptosis at both the early (39%) and late (16%) stages of apoptosis (Figure 4(a)). Confocal images clearly demonstrated that the translocation of phosphatidylserine (PS) from the inner leaflet of the plasma membrane to the cell surface, an early feature of cellular apoptosis, was induced by GTE treatment (Figure 4(b)). Additionally, fragmented and condensed nuclei were also identified by fluorescence microscopy with DAPI staining (Figure 4(c)). These observations suggest that GTE induces cell apoptosis in H23/0.3 cells.

### 3.5. GTE Activates the Mitochondria/Caspase Pathway

**Figure 5:**
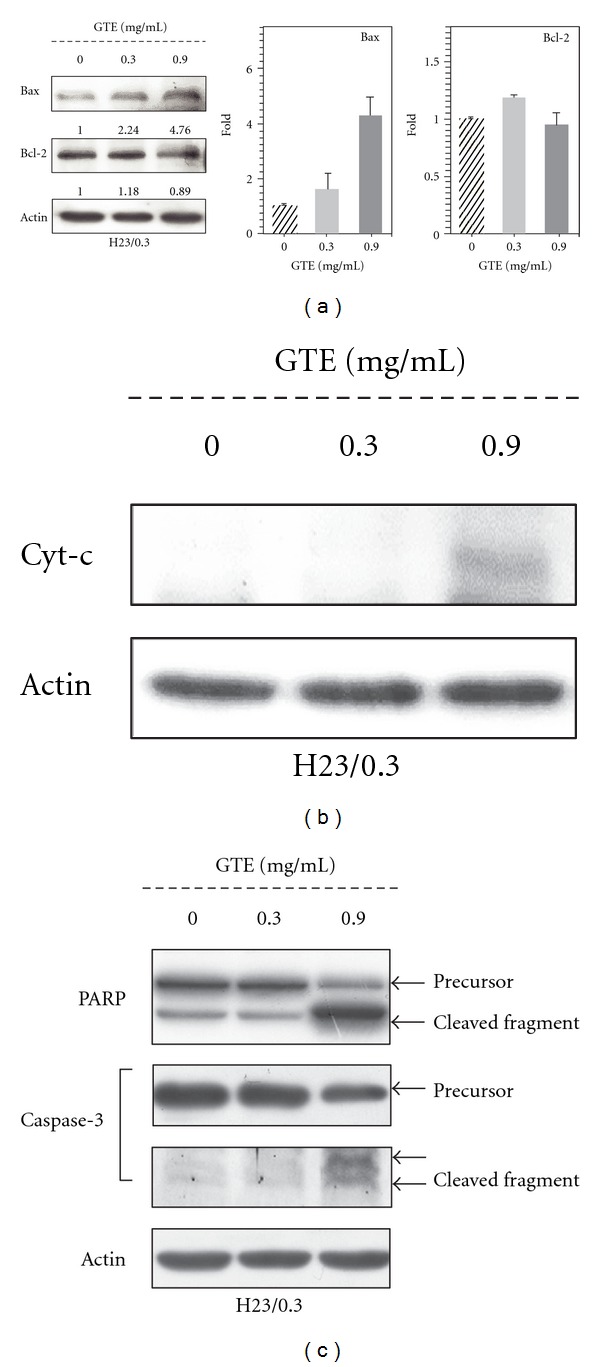
Effect of GTE on the mitochondria/caspase pathway in H23/0.3 cells. (a) H23/0.3 cells were treated with various concentrations of GTE (0 mg/mL, 0.3 mg/mL, and 0.9 mg/mL) for 24 h. The protein expression of Bcl-2 and Bax was measured by western blotting. (b) H23/0.3 cells were treated with various concentrations of GTE (0 mg/mL, 0.3 mg/mL, and 0.9 mg/mL) for 24 h. The release of cytochrome c (Cyt-c) into the cytoplasm was detected by western blotting. (c) H23/0.3 cells were treated with various concentrations of GTE (0 mg/mL, 0.3 mg/mL, and 0.9 mg/mL) for 24 h. The cleavage of caspase-3 and PARP was detected by western blotting. Results are expressed as the mean ± SD of three independent experiments.

To determine the underlying molecular mechanisms that are responsible for GTE-induced cell apoptosis, we examined the influence of GTE on the protein levels of members of the Bcl-2 family that mediate the activation of the mitochondria/caspase pathway [23]. As shown in Figure 5(a), GTE treatment resulted in a marked increase in the protein level of the proapoptotic protein Bax; however, treatment did not cause significant changes in the protein expression of the antiapoptotic protein Bcl-2. Moreover, we examined the effect of GTE on the release of Cyt-c and found that GTE caused a significant increase of Cyt-c protein in the cytosolic fractions (Figure 5(b)). In addition, treatment with GTE also caused a significant cleavage of caspase-3 and PARP (Figure 5(c)). These results demonstrate that GTE induces cell apoptosis by activating the mitochondria/caspase pathway in H23/0.3 cells.

### 3.6. GTE Inhibits Cell Proliferation via the PI3K/Akt Signaling Pathway

**Figure 6:**
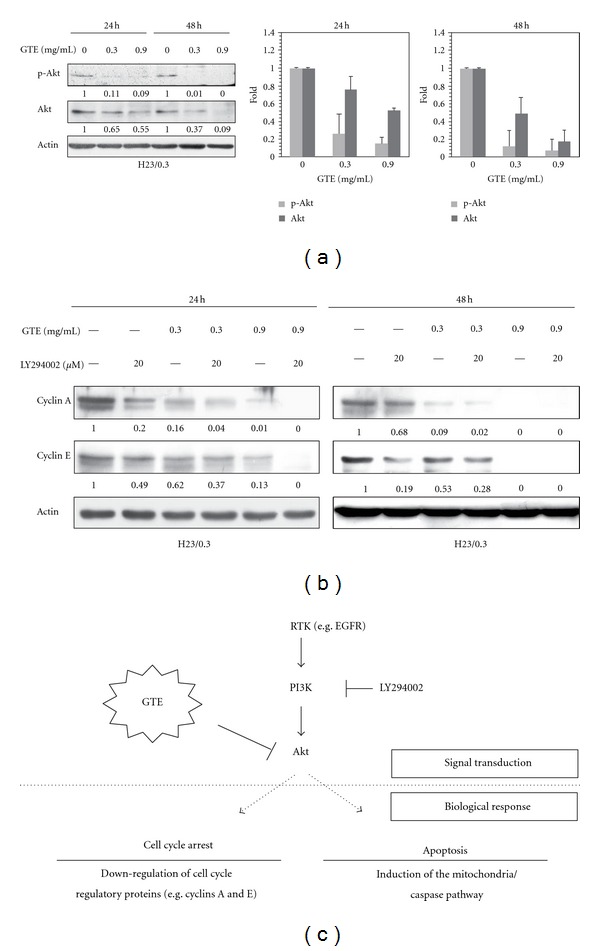
Effect of GTE on PI3K/Akt pathways in H23/0.3 cells. (a) H23/0.3 cells were treated with various concentrations of GTE (0 mg/mL, 0.3 mg/mL, and 0.9 mg/mL) for 24 h and 48 h. The protein levels of p-Akt and Akt were measured by western blotting. (b) H23/0.3 cells were treated with 20 *μ*M LY294002 (a PI3K inhibitor) alone or in combination with GTE (0.3 mg/mL or 0.9 mg/mL) for 24 h and 48 h. The expression levels of proteins (cyclins A and E) were measured by western blotting. (c) A proposed model for the GTE-mediated antiproliferation of doxorubicin-resistant lung adenocarcinoma H23/0.3 cells. Results are expressed as the mean ± SD of three independent experiments.

The PI3K/Akt signaling pathway is associated with cell proliferation, survival, and drug resistance in lung cancer [24]; therefore, we analyzed the influence of GTE on the PI3K/Akt signaling pathway. As shown in Figure 6(a), GTE exhibited dose- and time-dependent inhibitory effects on the levels of phospho-Akt and Akt. We next tested the validity of our results by incubating H23/0.3 cells with the PI3K inhibitor LY294002. The results revealed that LY294002 downregulated not only the protein levels of cyclin A but also the expression of cyclin E protein in H23/0.3 cells (Figure 6(b)). In other words, LY294002 exhibited an inhibitory effect that was similar to that of GTE on H23/0.3 cells. These data indicate that GTE inhibits cell proliferation by inhibiting the PI3K/Akt signaling pathway in H23/0.3 cells.

### 3.7. GTE Inhibits the Growth of H23/0.3 Xenografted Tumors

**Figure 7:**
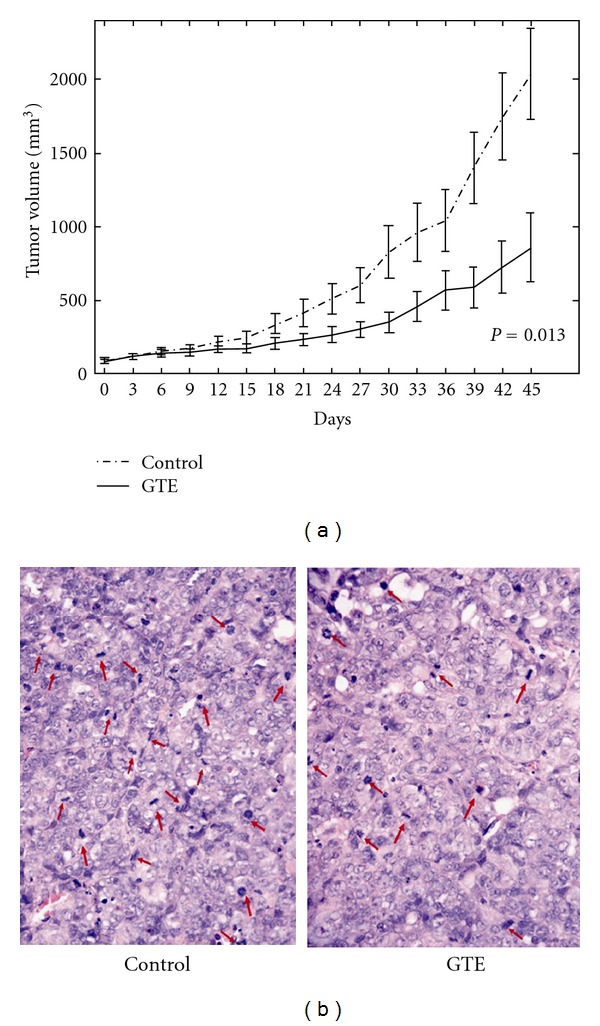
Effect of GTE on the growth of H23/0.3 xenografted tumors *in vivo*. (a) The tumor growth rate was significantly retarded in the GTE-treated group (100 mg/kg/day). The tumor volumes were estimated from the caliper measurements of three dimensions of the tumor. The estimated tumor volumes were calculated as *L* × *W* ^2^ × 0.5, where *L* is the major axis and *W* is tumor width. The data are represented as the mean ± SE (*n* = 10). (b) The tumor sections from the control mice showed more mitotic cells compared to those from the GTE-treated mice. The red arrows indicate mitotic figures in the H&E-stained areas (400X).

To verify the *in vivo* antitumor effect of GTE, we used xenografted tumor-bearing nude mice to examine the differences in tumor growth with and without GTE treatment. After the volume of the H23/0.3 xenografted tumor reached approximately 100–200 mm^3^, the nude mice were treated with either GTE (100 mg/kg/day) or vehicle orally (p.o.) for 45 days. As shown in Figure 7(a), the nude mice treated with GTE exhibited a significant suppression in H23/0.3 tumor growth relative to that of the control group. There was no significant difference in the body weights of the mice with and without GTE treatment (data not shown). In addition, as illustrated in Figure 7(b), we found that the numbers of mitotic cancer cells were significantly suppressed by the GTE treatment compared to those of the control group (85.43 ± 21.68 versus 58.71 ± 12 in 5 HPFs, *P* = 0.048). These results indicate that GTE suppresses the growth of H23/0.3 xenografted tumors *in vivo*.

We also evaluated the possibility of a toxic effect of GTE by conducting a pathological examination of all of the sacrificed and dissected mouse organs; the brain, heart, lung, liver, spleen, kidney, and intestines from 7 cases with and 7 cases without GTE treatment were examined. Toxicity was evaluated in terms of the severity of hemorrhage, necrosis, and inflammation on microscopic visual scales from “−” to “+++” that stand for none, mild, moderate, and severe, respectively. There were no significant differences in the aforementioned organ toxicities between the controls and the GTE-treated mice (Table 1).

## 4. Discussion

A number of Chinese herbal medicines have demonstrated significant potential as anticancer therapeutic agents due to their growth suppression effects on tumor cells [25, 26]. Among these medicines, *Ganoderma* is the most widely used herbal medicine in Asia and has been used for centuries. *Ganoderma tsugae* (GT), one of the most major species cultivated in Taiwan, has been shown to exhibit anti-proliferative effects against human tumor cells [12, 13]. In this study, we investigated the anticancer effects of the GT on the NSCLC H23/0.3 cells* in vitro* (Figure 2(a)) and *in vivo *(Figure 7(a)).

Botanical products, especially TCMs, are mixtures of phytochemicals that are not defined by the standard formula as pure chemical compounds, thus presenting a difficult issue for the scientific requirements of quality control (QC). Chemical fingerprinting is usually viewed as a gold standard to identify TCMs [27]; however, this method may not be the best method to achieve this core purpose. In comparison with the classification of botanical products by chemical fingerprints, the biological response will be more appropriate in characterizing the therapeutic effects, particularly for those products with mixed compounds, such as TCM [16]. In this study, we used a comprehensive PhytomicsQC platform as a scientific approach for the QC of TCMs (e.g., GTE). Both the bioresponse fingerprint and the chemical fingerprint [16] of GTEs were determined and revealed the high quality and consistency among different batches of GTE (Figures 1(a)–1(c)).

Perturbation of progression of the cell cycle in tumor cells is a helpful strategy to halt tumor growth [21]. Furthermore, the cell cycle arrest of tumor cells also provides an occasion for cells to undergo either repair or cellular apoptosis. A number of TCMs exhibit significant inhibitory effects on lung cancer cells via disruption of cell cycle progression and/or induction of cell apoptosis [25, 26]. Previous reports showed that a G2/M cell cycle arrest was induced in Hep3B cells treated with the chloroform extract of GT [9] and in COLO 205 cells exposed to the methanol extract of GT [10]. In this study, our *in vitro* data indicated that the treatment of H23/0.3 cells with ethanol extract of GT induced S phase arrest (Figure 3(a)) via modulation of the expression of S phase regulatory proteins (Figures 3(b) and 3(c)) in H23/0.3 cells. The various effects of GT on the distribution of cell cycle may be due to cell-type specificity and/or result from variations in preparation process of GT extract.

Many anticancer drugs/agents exert their anticancer activities by inducing the cellular apoptosis of tumor cells [28]. Resistance to cellular apoptosis, therefore, results in a decrease in the sensitivity of cancer cells to drugs and the failure of chemotherapy [22, 29]. Several TCMs have been reported to induce cellular apoptosis in lung cancer [25, 26]. For example, *Typhonium blumei* extract induces cellular apoptosis via the mitochondrial/caspase pathway by upregulating the expression of proapoptotic proteins (e.g., Bax, Bad, and Bak), downregulating the expression of anti-apoptotic proteins (e.g., Bcl-2 and Bcl-xL), and activating caspase-9 and caspase-3 in lung cancer A549 cells [26], whereas *Scutellaria baicalensis* extract induces apoptosis by upregulating the expression of the proteins p53 and Bax in lung cancer A549 cells [25]. In this study, our results indicated that GTE not only perturbed cell cycle progression but also induced cellular apoptosis in lung cancer H23/0.3 cells (Figures 4(a)–4(c)). Furthermore, we also found that the treatment of H23/0.3 cells with GTE resulted in a marked increase in the expression of Bax (Figure 5(a)). This increase may be responsible for the concomitant execution phase of cellular apoptosis such as an increase in the release of Cyt-c from the mitochondria to the cytosol and the activation/cleavage of caspase-3 and PARP (Figure 5(c)).

The PI3K/Akt signaling pathway plays a critical role in cell proliferation, survival, and drug resistance in lung cancer [24]. Therefore, the suppression of the PI3K/Akt signaling pathway may be an effective approach to the treatment of lung cancer [30, 31]. In this study, we found that GTE inhibits the protein levels of Akt and phospho-Akt in H23/0.3 cells (Figure 6(a)). The inhibitory influence of GTE on phospho-Akt levels may be governed by its ability to suppress the expression of the Akt protein. We incubated H23/0.3 cells with LY294002, a PI3K-specific inhibitor, to confirm that GTE inhibits the proliferation of lung cancer H23/0.3 cells via modulation of the PI3K/Akt signaling pathway (Figure 6(b)). These results suggest that GTE may be a useful Akt-targeting agent for the treatment of lung cancer.

A number of reports show that the combined usage of some extracts from herbs (such as coptis rhizoma and glycyrrhizae radix) with anticancer agents results in a synergistic growth inhibitory effect on cancer cells [32, 33]. It has also been reported that a combination of *Ganoderma* with anticancer agents significantly slows the growth rate of cancer cells [13, 34]. For example, the combined treatment of taxol with *Ganoderma tsugae* (GT) extract results in a synergistic growth suppression effect on colorectal cancer COLO205 cells [13], and *Ganoderma lucidum* enhances the chemotherapeutic efficacy of doxorubicin against SCLC H69 and VPA cells (a multidrug resistant cell line derived from H69 cells) [34]. Similarly, we demonstrated that a combination of doxorubicin with GTE resulted in a marked reduction in the number of viable NSCLC H23/0.3 cells (Figure 2(c)). These results suggest that GTE may be a promising adjuvant to anticancer agents in the treatment of drug resistant NSCLC cells.

In conclusion, we have demonstrated that GTE induces S phase arrest and the cellular apoptosis of H23/0.3 cells via regulation of the PI3K/Akt signaling pathways (Figure 6(c)). In addition, we have also shown that a combination of GTE and doxorubicin exerts an enhanced growth inhibitory effect on H23/0.3 cells. Our results suggest that GTE may be a safe and effective adjuvant therapeutic agent for the treatment of NSCLC cells with drug resistance.

## Figures and Tables

**Table 1:** A comparison of the organ toxicity of the control and GTE treatment.

	Heart			Lung			Kidney			Brain			Liver			Spleen			Intestines		
	H	N	I	H	N	I	H	N	I	H	N	I	H	N	I	H	N	I	H	N	I
A1	*−*	*−*	*−*	++	*−*	*−*	+	*−*	*−*	*−*	*−*	*−*	+	*−*	*−*	+	*−*	*−*	*−*	*−*	*−*
A2	*−*	*−*	*−*	+	*−*	*−*	+	*−*	*−*	*−*	*−*	*−*	+	*−*	*−*	+	*−*	*−*	*−*	*−*	*−*
A3	+	*−*	*−*	++	*−*	*−*	+	*−*	*−*	*−*	*−*	*−*	+	*−*	*−*	+	*−*	*−*	*−*	*−*	+
A4	+	*−*	*−*	+++	*−*	*−*	+	*−*	*−*	*−*	*−*	*−*	+	*−*	*−*	+	*−*	*−*	*−*	*−*	*−*
A5	+	*−*	*−*	++	*−*	*−*	*−*	*−*	*−*	*−*	*−*	*−*	+	*−*	*−*	+	*−*	*−*	*−*	*−*	*−*
A6	++	*−*	*−*	++	*−*	*−*	+	*−*	*−*	*−*	*−*	*−*	+	*−*	*−*	+	*−*	*−*	*−*	*−*	+
B1	+	*−*	*−*	++	*−*	*−*	+	*−*	*−*	*−*	*−*	*−*	++	*−*	*−*	+	*−*	*−*	*−*	*−*	+
B2	++	*−*	*−*	+++	*−*	*−*	+	*−*	*−*	*−*	*−*	*−*	+	*−*	*−*	+	*−*	*−*	*−*	*−*	*−*
B3	*−*	*−*	*−*	++	*−*	*−*	*−*	*−*	*−*	*−*	*−*	*−*	+	*−*	*−*	+	*−*	*−*	*−*	*−*	+
B4	+++	*−*	*−*	++	*−*	*−*	+	*−*	*−*	*−*	*−*	*−*	+	*−*	*−*	+	*−*	*−*	*−*	*−*	*−*
B5	+	*−*	*−*	+++	*−*	*−*	*−*	*−*	*−*	*−*	*−*	*−*	+	*−*	*−*	+	*−*	*−*	*−*	*−*	*−*
B6	++	*−*	*−*	+	*−*	*−*	+	*−*	*−*	*−*	*−*	*−*	+	*−*	*−*	+	*−*	*−*	*−*	*−*	*−*
B7	+	*−*	*−*	++	*−*	*−*	+	*−*	*−*	*−*	*−*	*−*	+	*−*	*−*	+	*−*	*−*	*−*	*−*	*−*
C1	+	*−*	*−*	++	*−*	*−*	+	*−*	*−*	*−*	*−*	*−*	+	*−*	*−*	+	*−*	*−*	*−*	*−*	+
C2	+	*−*	*−*	+++	*−*	*−*	+	*−*	*−*	*−*	*−*	*−*	+	*−*	*−*	+	*−*	*−*	*−*	*−*	+
C3	+	*−*	*−*	++	*−*	*−*	+	*−*	*−*	*−*	*−*	*−*	+	*−*	*−*	+	*−*	*−*	*−*	*−*	*−*
C4	+	*−*	*−*	++	*−*	*−*	+	*−*	*−*	*−*	*−*	*−*	+	*−*	*−*	+	*−*	*−*	*−*	*−*	*−*
C5	+++	*−*	*−*	+++	*−*	*−*	+	*−*	*−*	*−*	*−*	*−*	+	*−*	*−*	+	*−*	*−*	*−*	*−*	+
C6	+	*−*	*−*	+++	*−*	*−*	*−*	*−*	*−*	*−*	*−*	*−*	+	*−*	*−*	+	*−*	*−*	*−*	*−*	*−*
C7	*−*	*−*	*−*	++	*−*	*−*	+	*−*	*−*	*−*	*−*	*−*	+	*−*	*−*	+	*−*	*−*	*−*	*−*	*−*

A1–6: mice that received neither a tumor implant nor GTE treatment; B1–7: mice that received a tumor implant but did not receive GTE treatment; C1–7: mice that received both a tumor implant and GTE treatment; H: hemorrhage; N: necrosis; I: Inflammation. The severity of the visual scoring system represented by “−” and “+”; − stands for none, + stands for minimal, ++ stands for visible, and +++ stands for apparent pathological change.
